# Modeling sunset yellow removal from fruit juice samples by a novel chitosan-nickel ferrite nano sorbent

**DOI:** 10.1038/s41598-023-50284-0

**Published:** 2024-01-02

**Authors:** Samira Shokri, Nabi Shariatifar, Ebrahim Molaee-Aghaee, Gholamreza Jahed Khaniki, Parisa Sadighara, Mohammad Ali Faramarzi

**Affiliations:** 1 Department of Environmental Health Engineering, Food Safety Division, School of Public Health, Tehran University of Medical Sciences, Tehran, Iran; 2 Department of Pharmaceutical Biotechnology, Faculty of Pharmacy, Tehran University of Medical Sciences, Tehran, Iran

**Keywords:** Environmental sciences, Chemistry

## Abstract

Analysis of food additives is highly significant in the food industry and directly related to human health. This investigation into the removal efficiency of sunset yellow as an azo dye in fruit juices using Chitosan-nickel ferrite nanoparticles (Cs@NiFe_2_O_4_ NPs). The nanoparticles were synthesized and characterized using various techniques. The effective parameters for removing sunset yellow were optimized using the response surface methodology (RSM) based on the central composite design (CCD). Under the optimum conditions, the highest removal efficiency (94.90%) was obtained for the initial dye concentration of 26.48 mg L^−1^ at a pH of 3.87, a reaction time of 67.62 min, and a nanoparticle dose of 0.038 g L^−1^. The pseudo-second-order kinetic model had a better fit for experimental data (R^2^ = 0.98) than the other kinetic models. The equilibrium adsorption process followed the Freundlich isotherm model with a maximum adsorption capacity of 212.766 mg g^−1^. The dye removal efficiency achieved for industrial and traditional fruit juice samples (91.75% and 93.24%), respectively, confirmed the method's performance, feasibility, and efficiency. The dye adsorption efficiency showed no significant decrease after five recycling, indicating that the sorbent has suitable stability in practical applications. variousThe synthesized nanoparticles can be suggested as an efficient sorbent to remove the sunset yellow dye from food products.

## Introduction

Food additives critically impact food safety and quality^1^. In the food industry, both natural and synthetic colours are frequently utilized^2^. Natural dyes are usually unstable and readily destroyed during food processing. Therefore Synthetic dyes with chemical compounds similar to natural colorants are extensively utilized in food products^3^.

Azo dyes, the largest group of synthetic food colorants, constitute 70% of all organic dyes produced worldwide^4^. They are decomposed and converted into secondary amine metabolites called benzidine due to the activity of microorganisms and heat, which are often more stable and resistant to oxidizing agents, pH variations, heat, UV radiation, acids, and alkalis in terms of their complex chemical structure^3,5,6^. Toxicological studies indicated that azo dyes could cause mild to severe side effects, such as persistent headaches, hyperactivity in children, asthma, allergic reactions, urticarial, liver and kidney disorders, cancer, and interference with human serum albumin and hemoglobin^7–10^.

The list of permitted food colorants varies from country to country^11–13^. The permitted synthetic food dyes in Iran include sunset yellow (orange), quinoline yellow (yellow), brilliant blue (blue), indigotin or indigo carmine (blue), Allura red (red), ponceau (red), and carmoisine (red). However, they have been prohibited in some products, e.g., ice cream, drinks, fruit juice, desserts, and traditional products^14^.

Sunset Yellow, a famous synthetic food dye, is a hydroxyl mono-azo disulfone dye with conjugated aromatic rings and an azo functional group (N = N)^13^. The maximum daily intake of 4 mg per kilogram of body weight has been determined for this colorant by the World Health Organization (WHO) and the Food and Agriculture Organization (FAO)^15,16^. Its maximum allowed level in alcoholic and non-alcoholic beverages is 200 and 50 mg L^−1^, respectively^16^.

Two widely used methods for removing dyes as contaminants from food sources include the adsorption process in the presence of a sorbent and photodegradation in the presence of a photocatalyst^17,18^. The adsorption process involves binding contaminant molecules to a solid material, known as a sorbent, resulting in their removal from real samples^19–22^. This method can be tailored to target specific dye molecules, making it versatile and precise. It effectively removes a wide range of synthetic dyes commonly used in the food industry and is cost-effective and environmentally friendly^23^. The procedure can be customized to different food matrices and is easily applicable to various types of food products^24^. Additionally, the reusability of sorbents makes the process economically viable and reduces waste generation, highlighting its environmental sustainability.

Nanostructured materials such as multilayers, nanowires, nanoparticles, and nanocomposites have attracted vast attention due to their specific optical, electrical, chemical, and magnetic properties^25–30^. Simultaneous control of one or more parameters during food production or processing, transportability, and high speed and accuracy evaluation are among the characteristics of nanocomposites^31^. Spinel ferrites with excellent magnetic and electronic properties and reusability have been interesting magnetic oxide nanoparticles in the last decades^32,33^. Nickel ferrite, a soft semiconductor spinel ferrite, has high electrical resistance, and low magnetic saturation^34,35^. For biomedical applications, nanoparticles should be coated with biocompatible polymers like chitosan to ensure stability, biodegradability, and non-toxicity in the physiological environment^36–38^. Chitosan, a natural polymeric sorbent, has been reported to eliminate anionic dyes from solutions^39^.

The limitation of chitosan is its difficult separation from the final solution. The chitosan-magnetic nanoparticle composites can easily be separated from the solution via an external magnet^40^.

This research evaluated the adsorption rate of sunset yellow on chitosan-coated nanoparticles. A two-level CCD was utilized to optimize the parameters effective on sunset yellow removal, including sorbent amount, reaction time, dye concentration, and pH. The experimental design reduces the number of tests, costs, and time in the process of optimizing the useful components via the simultaneous examination of several parameters. The adsorption behaviour was examined using pseudo-first-order, pseudo-second-order, and intraparticle diffusion kinetic models, as well as Langmuir and Freundlich isotherms.

## Materials and methods

### Materials

Chitosan (Low Mw, 50,000–190,000 KDa), NaOH, NiCl_2_ .6H_2_O, FeCl_3_ .6H_2_O, oleic acid, acetic acid (˃ 98%), and sunset yellow (98%) were supplied by Merck Company (Darmstadt, Germany).

### Synthesis of NiFe_2_O_4_ nanoparticles

Initially, a solution of NiCl_2_·6H_2_O (0.2 M, 20 mL) was combined with a solution of FeCl_3_.6H_2_O (0.4 M, 20.0 mL) under stirring at room temperature. The pH of the solution was adjusted to approximately 13 using an aqueous solution of NaOH (3.0 M). The mixture was stirred at 60 °C for 30 min, followed by the addition of 0.01 mL of oleic acid to the solution as a surfactant. The mixture was then heated at 80 °C for 40 min. The resulting brown precipitates (amorphous NiFe_2_O_4_ NPs) were separated using a magnet, subsequently rinsed with deionized water and ethanol several times, and finally dried at 80 °C for 2–3 h. The resulting amorphous NiFe_2_O_4_ nanoparticles were sealed in a stainless-steel autoclave with Teflon coating and kept at 600 °C for 6 h^41–45^.

### Synthesis of Cs@NiFe_2_O_4_ NPs

**Figure 1:**
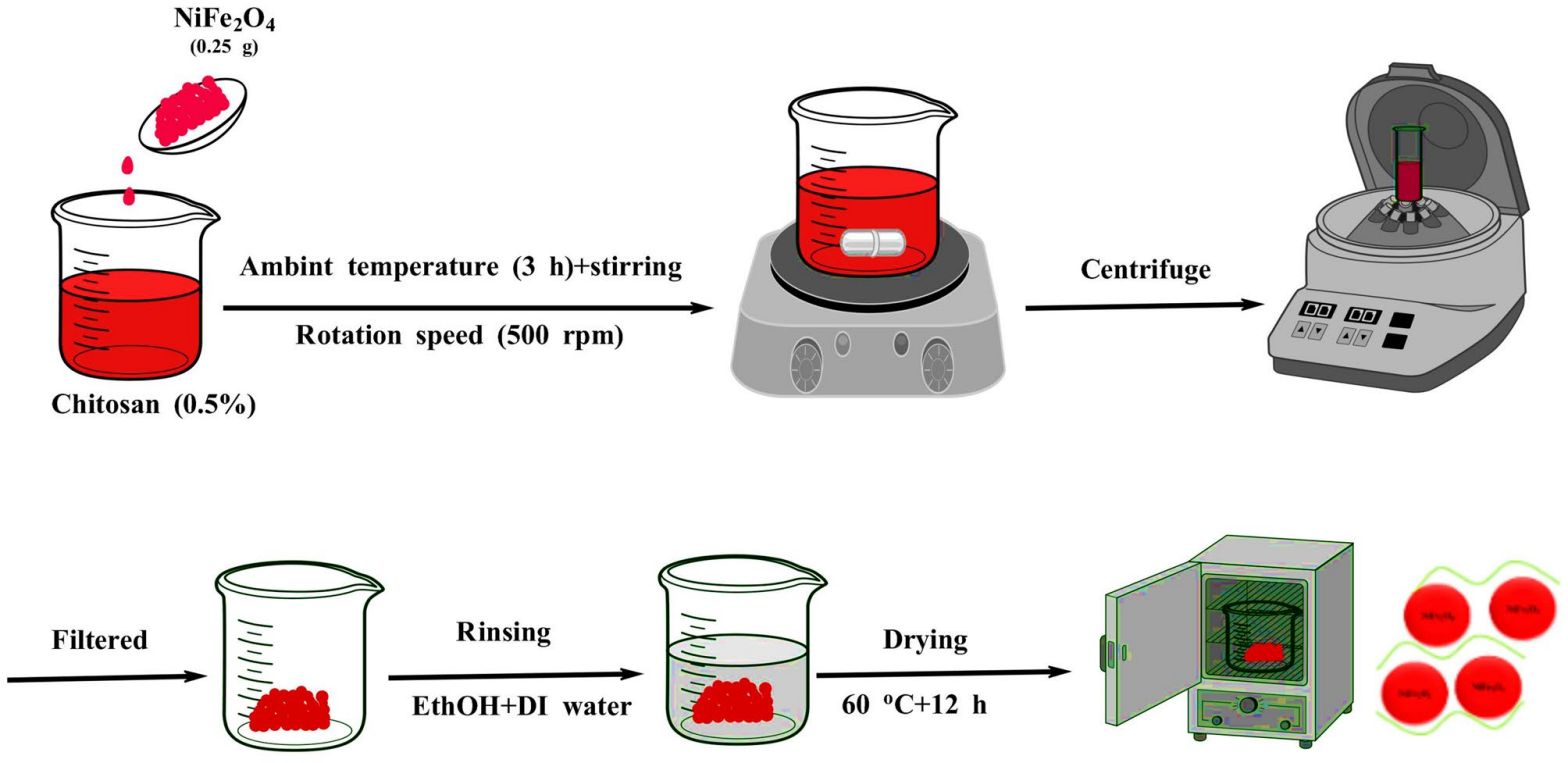
The schematic image of the adorbent synthesis procedure.

Chitosan solution was prepared by dissolving 1 g of chitosan with 50.0 mL of 1% acetic acid solution. Subsequently, 0.25 g of NiFe_2_O_4_ NPs were added to this solution. The suspension was stirred at ambient temperature for 3 h at 500 rpm. Following this, the mixture was centrifuged to separate the resulting brown precipitates (CS@NiFe_2_O_4_ NPs), and then rinsed with ethanol and distilled water before being dried at 60 °C for 12 h^41,42^. The schematic image of the adsorbent synthesis method is presented in Fig. 1.

### Characterization

Fourier transform infrared spectroscopy (FTIR) was achieved using Bruker Vertor-22 FTIR with a resolution of 4 cm^−1^ in the 500–4000 cm^−1^ range. At 25 °C, the X-Ray diffraction (XRD) pattern was captured at 2θ = 5–80° using a 0.04°/min scanning speed. A transmission electron microscope (TEM, EM Philips EM 208S) was employed to observe the particle size and dispersibility of the composite nanoparticles. Using scanning electron microscopy (SEM, MIRA3-LUM, Czech), the morphological properties were also studied, and the specific surface area was found by the Brunauer–Emmett–Teller (BET) Surface Area & Porosity Analyzer (II BELSORP mini, Japan). The mass loss of the materials was investigated with a thermogravimetric analyzer (TGA; TGA STA6000, PerkinElmer Corporation, USA) ranging from 20 to 850 °C at a heating rate of 10 °C/min using nitrogen as purge and protective gas.

### Experiment design and optimization

The RSM method was used to design sorption experiments using Design Expert software. This model helps to describe the effect of the influencing factors and interrelationships with the least number of experiments considering optimal values for each factor. A total of 30 experiments were determined according to preliminary tests (Table 1). Data analysis for the desired response, the removal efficiency, was performed considering the confidence interval of 95%.

**Table 1:** The principal factors, experiments, and responses obtained in the CCD for sunset yellow removal by Cs@NiFe_2_O_4_ NPs.

Factor	Name	Unit	Minimum	Maximum	Mean ± SD
F_1_	Dye concentration	mg L^−1^	10.00	50.00	30.00 ± 15.76
F_2_	Sorbent amount	g L^−1^	0.0200	0.0600	0.0400 ± 0.0158
F_3_	Contact time	min	20.00	70.00	45.00 ± 19.70
F_4_	pH		3.00	9.00	6.00 ± 2.36

Run	F_1_	F_2_	F_3_	F_4_	Response
	Dye concentration (mg L^−1^)	Sorbent amount (g L^−1^)	Contact time (min)	pH	Removal Efficiency (%)
1	10	0.02	20	3	79.48
2	10	0.04	45	6	74.08
3	50	0.02	70	9	50.46
4	30	0.06	45	6	80.58
5	50	0.06	20	3	74.32
6	50	0.02	20	9	51.08
7	30	0.04	45	9	64.64
8	50	0.02	20	3	77.65
9	30	0.04	45	6	88.63
10	30	0.04	20	6	76.11
11	30	0.04	45	6	91.59
12	10	0.06	70	9	72.54
13	30	0.04	45	6	94.61
14	10	0.02	70	3	78.46
15	10	0.06	20	3	58.18
16	50	0.06	70	3	88.93
17	30	0.02	45	6	76.62
18	30	0.04	45	6	90.96
19	30	0.04	45	6	93.28
20	10	0.02	70	9	53.24
21	10	0.06	70	3	87.76
22	30	0.04	70	6	92.09
23	50	0.02	70	3	70.68
24	30	0.04	45	3	87.56
25	10	0.02	20	9	46.86
26	30	0.04	45	6	87.64
27	50	0.04	45	6	80.19
28	50	0.06	20	9	48.72
29	50	0.06	70	9	86.68
30	10	0.06	20	9	39.56

To evaluate the sorption efficiency, the Cs@NiFe_2_O_4_ NPs were separated from the samples at the end of the process by centrifuging at 4000 rpm for 10 min. The sediments were removed using a magnet. The absorbance of the solution was calculated using a UV–visible spectrophotometer (Uviline 9400, Secomam) at a wavelength of 485 nm. The dye reduction percentage and the adsorption amount were calculated by Eqs. (1) and (2), respectively.

1 $$R\%=\frac{\left({C}_{0}-{C}_{e}\right)}{{C}_{0}}$$

2 $${q}_{e}=\frac{V}{M}\left({C}_{0}-{C}_{e}\right)$$

where R is the dye removal efficiency (%), C_0_ is the initial dye concentration (mg L^−1^), C_e_ is the equilibrium dye concentration (mg L^−1^), q_e_ is the adsorption capacity (mg g^−1^), V is the sample volume (L), and M is the sorbent mass (g L^−1^ )^46,47^.

### Adsorption isotherms

Adsorption isotherms, including Langmuir and Freundlich isotherms, were used to investigate the behavior of the synthesized sorbent and the interaction between the sorbent and the dye. Isotherm tests were performed at a pH of 3.84, an equilibrium time of 68 min, and various dye concentrations (10, 15, 20, 25, 30, 35, 40, 45, and 50 mg L^−1^)^46^.

### Models of the kinetics of adsorption

Intraparticle diffusion, pseudo-first-order, and pseudo-second-order kinetic models were used to control, quantitatively analyze, and determine the mechanism of the adsorption process of sunset yellow dye by the synthesized Cs@NiFe_2_O_4_ NPs. The adsorption kinetic parameters were calculated by matching equations obtained from charts with adsorption kinetic equations. Kinetic experiments were performed under varying contact times (10, 20, 30, 40, 50, 60, 70, and 80 min) at an initial dye concentration of 26.48 mg L^−1^ using the optimized sorbent dose (0.038 g L^−1^) in the pH of 3.8^46^.

### Real sample study

The application of the nanocomposites to remove sunset yellow dye in the orange juices purchased from a local market (Tehran, Iran) and traditional orange juices spiked with 5 mg L^−1^ of sunset yellow dye was investigated to evaluate the impact of the matrix on dye removal. It has been confirmed that all methods, the experimental data collection, and the plant experiments complied with relevant with relevant institutional, national, and international guidelines and legislation. The samples were placed in an ultrasonic bath for 60 min and then diluted with deionized water. Afterward, the samples were filtered with 0.45 μm membranes. Nanoparticles were added to the sample solution (0.038 g L^−1^), and after 68 min at 20 °C, they were removed with a magnet from the solution. The absorbance of the samples was measured before adding nanoparticles to the sample solution and after separating the nanoparticles with a spectrophotometer at 485 nm. The removal efficiency was calculated as follows Eqs. (1).

## Results and discussion

### FTIR analysis

**Figure 2:**
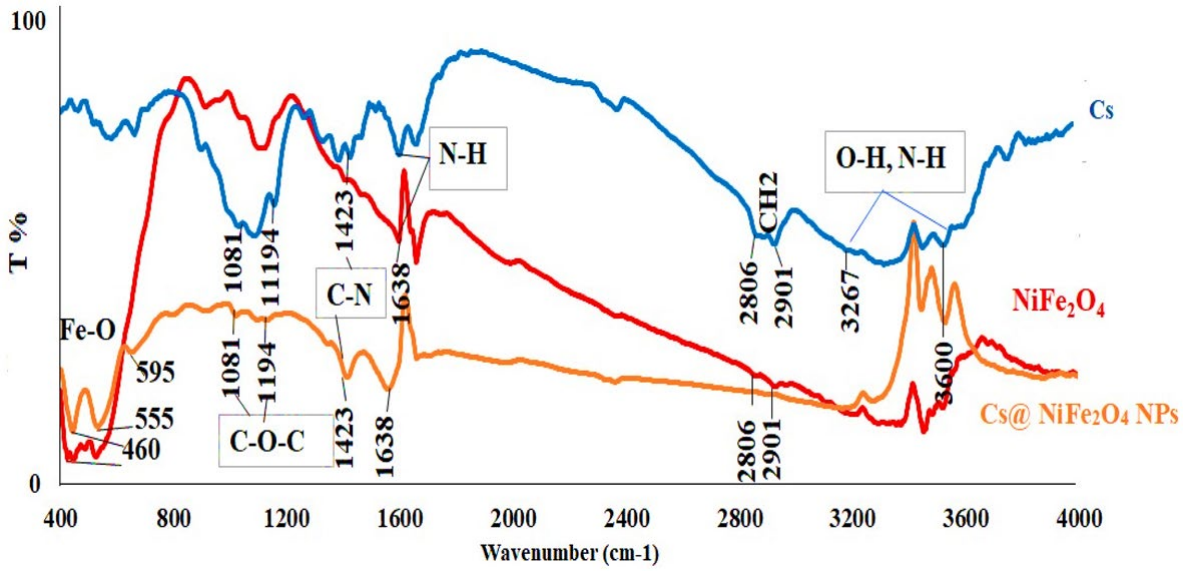
The FTIR spectra of Cs, NiFe_2_O_4_ NPs, Cs@NiFe_2_O_4_ NPs.

The FTIR spectra of Cs, NiFe_2_O_4_ NPs, and Cs@NiFe_2_O_4_ NPs are presented in Fig. 2. The FTIR spectrum of Cs indicates a broad band at 3600–3267 cm^-1^ attributed to O–H (hydroxyl group) and NH_2_ (amine group) starching vibrations^48,49^, sharp peaks owing to symmetric and asymmetric C–H stretching vibrations at 2901 cm^−1^ and 2806 cm^−1^, respectively, and peaks at 1638, 1423, and 1194 cm^-1^ due to N–H (amide I) bending, C–N carboxylic vibrations of the glycoside ring, C–O–C respectively^50,51^.

The FTIR pattern of NiFe_2_O_4_ NPs demonstrated a characteristic band in 460–595 cm^−1^ related to the Fe–O stretching vibration. The sharp band in 1764 cm^−1^ can be assigned to the vibrations of ions of sulfate adsorbed on the particle surface of NiFe_2_O_4_^49,52^.

Similar peaks are also observed in the FTIR range of Cs@NiFe_2_O_4_ NPs. The only difference is slight variations in the peaks’ appearance and intensity. The results confirmed the successful coating of NiFe_2_O_4_ nanoparticles by chitosan, probably through chemical reaction and electrostatic interaction between the chitosan's positively charged surface and the nickel ferrite's negatively charged surface.

### XRD analysis

**Figure 3:**
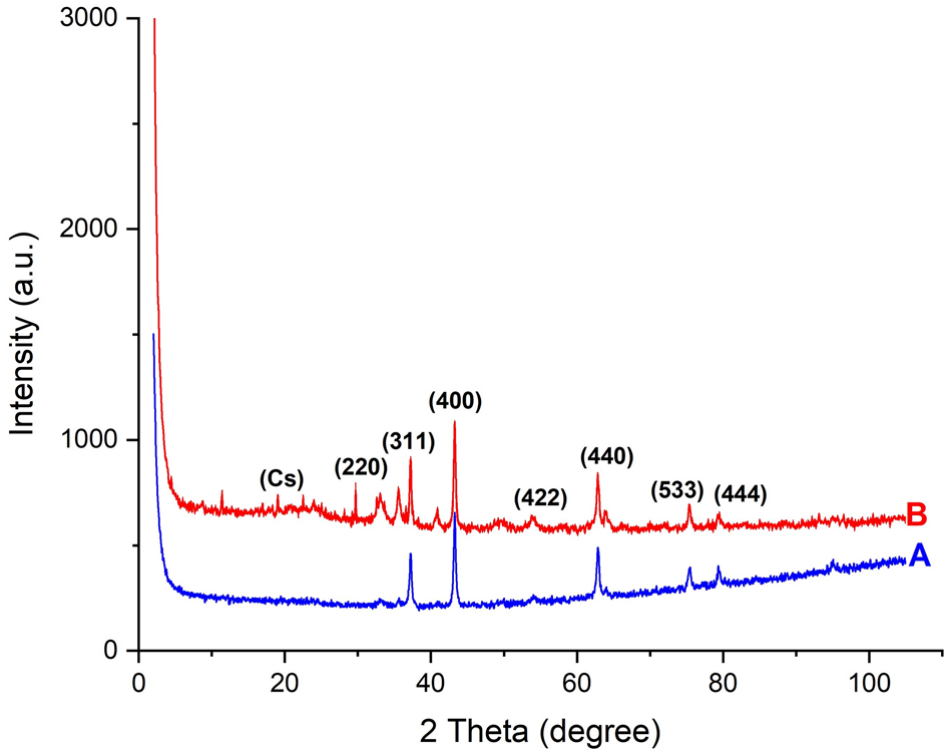
The XRD patterns of NiFe_2_O_4_ NPs (**A**) and Cs@NiFe_2_O_4_ NPs (**B**).

In Fig. 3A and B, the XRD patterns show NiFe_2_O_4_ NPs with and without chitosan coating.

The peaks at 2θ = 30.12°, 37.23°, 43.26°, 54.32°, 63.85°, 75.40°, and 80.02°, respectively, indicate the crystal planes (222), (311), (400), (422), (440), (533), and (444) (Fig. 3A). NiFe_2_O_4_ nanoparticles' XRD pattern matches the XRD spectrum on JCPDS standard card no. 10-0325^53,54^.

The XRD pattern of Cs@NiFe_2_O_4_ NPs also revealed distinctive NiFe_2_O_4_ NPs peaks (Fig. 3B). The retention of the same characteristic peaks and the unchanged intensity of the peaks after the activation of NiFe_2_O_4_ NPs coated by Cs composite implies an ideal compatibility and good interaction with the preservation of the crystal structure of nanoparticles at a high level.

Also there are peaks at 2θ = 20.50° and  10.32°, that are identical to pure chitosan in terms of strength and location^50^. The chitosan peaks in the XRD pattern of Cs@NiFe_2_O_4_ NPs also exhibit a low intensity. It can be explained by how interactions with different monomers cause chitosan's well-crystalline linear structure to be disrupted. These findings supported the effective coating of Cs on nanoparticles.

### TGA analysis

**Figure 4:**
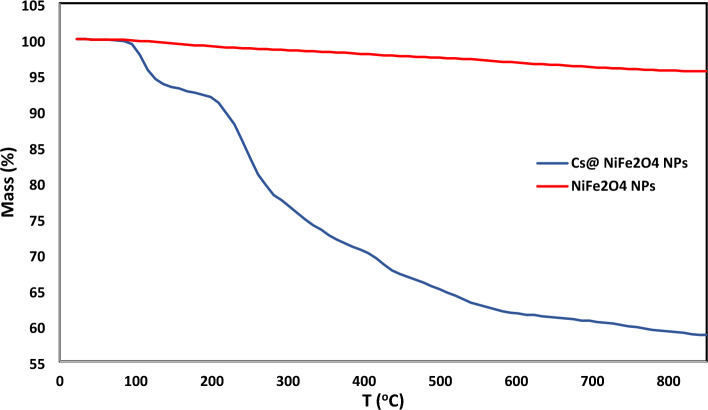
The TGA of NiFe_2_O_4_ NPs and Cs@NiFe_2_O_4_ NPs.

The sorbent's thermal stability was examined utilizing thermogravimetric analysis (Fig. 4). The graph illustrates the high stability of NiFe_2_O_4_ NPs up to 800 °C, with minimal degradation of approximately 5%. However, upon heating the sorbent (Cs@NiFe_2_O_4_ NPs), a decrease in mass is observed at 197 °C, attributed to the removal of water molecules, indicating that the weight percentage of moisture in the absorbent is approximately 8%. Subsequently, a sharp decline in the weight percentage of the sorbent is evident as the temperature increases to 800 °C, signifying the degradation of chitosan. This observation reveals a decrease in weight percentage of 29% at this stage, indicating the percentage of chitosan present in the sorbent.

### SEM analysis

**Figure 5:**
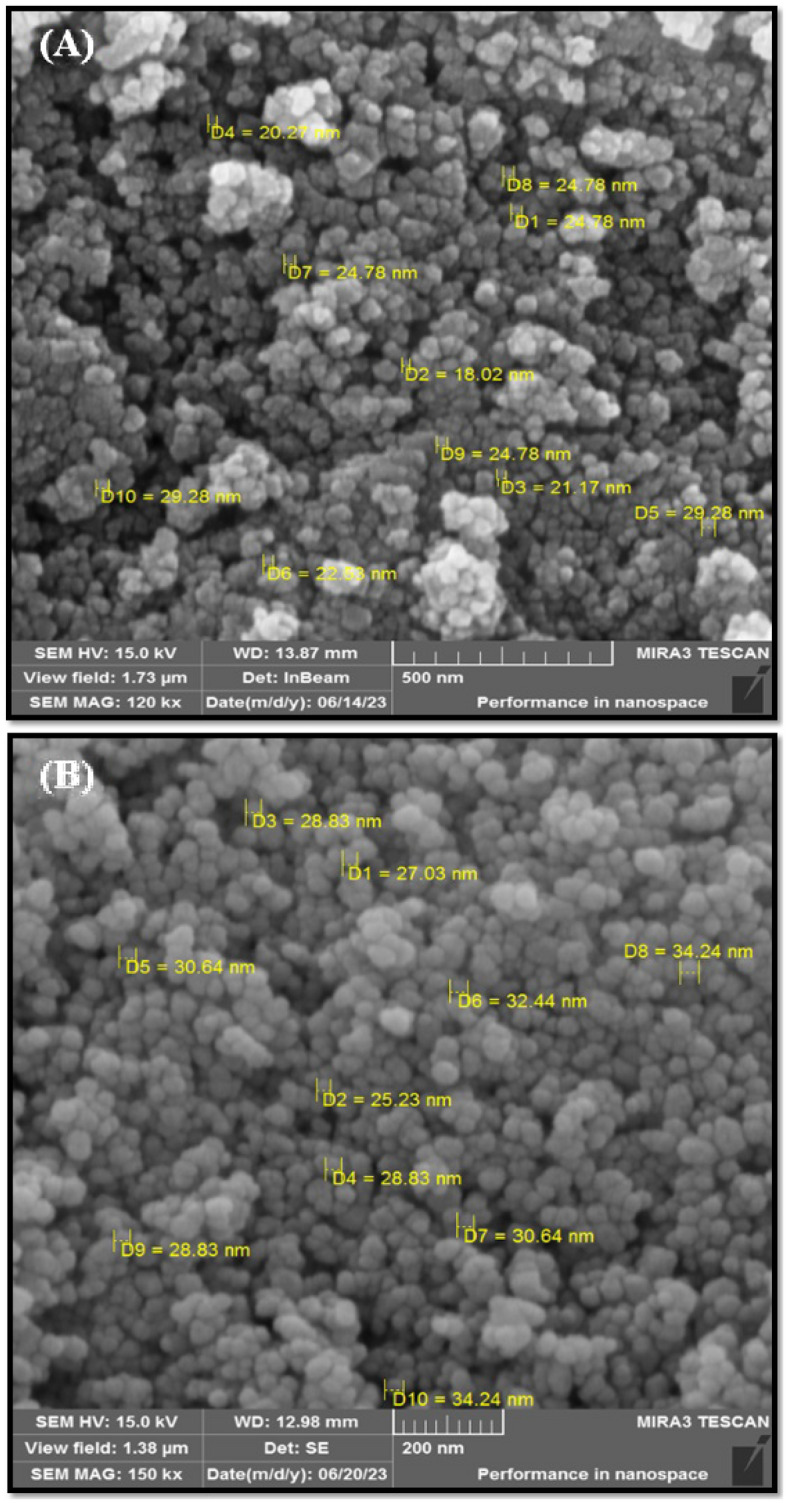
The SEM images of NiFe_2_O_4_ NPs (**A**) and Cs@NiFe_2_O_4_ NPs (**B**).

The SEM pictures of uncoated and chitosan-coated nickel-ferrite nanoparticles are presented in Fig. 5. The NiFe_2_O_4_ NPs (Fig. 5A) demonstrated a high accumulation, relatively large holes, and heterogeneous surface structures. Chitosan-coated nickel ferrite (Fig. 5B) is composed of spherical particles. The average particle size is in the nanoscale, and the largest particles have a diameter of approximately 34 nm. The precipitation rate of the intermediate product is higher than the rate of surface agent arrangement on the particles. After the initial precipitation, chitosan is adsorbed on the particles and declines the surface tension, turning them into spheres with an appropriate surface-to-volume ratio, which leads to the formation of well-differentiated nanospheres with floatability. These findings indicate how chitosan works to keep particles from sticking together and confirm the coating of particles by chitosan. The results were consistent with others^55,56^.

### TEM analysis

**Figure 6:**
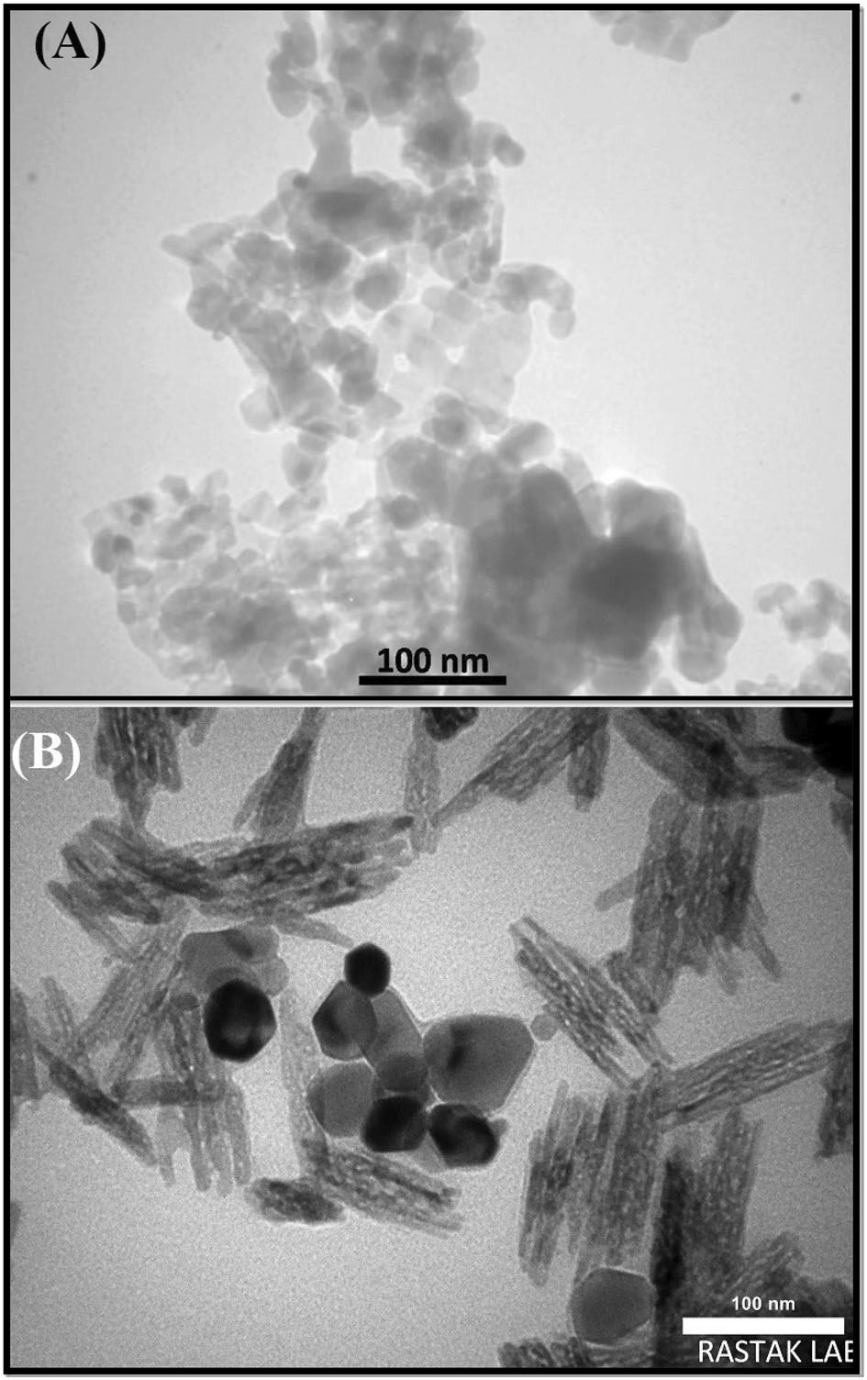
The TEM images of NiFe_2_O_4_ NPs (**A**) and Cs@NiFe_2_O_4_ NPs (**B**).

The internal structure and microstructural characteristics of NiFe_2_O_4_ and Cs@NiFe_2_O_4_ NPs were analyzed by TEM (Fig. 6). The NiFe_2_O_4_ nanoparticles (Fig. 6A) showed spherical or elliptical shape with various sizes that aggregated due to strong magnetic dipole interactions.

The incorporation of Cs into NiFe_2_O_4_ NPs changed the surface morphology and roughness. The TEM image (Fig. 6B) indicates that the rod-shaped particles are a result of the formation of chitosan nanoparticles within the sorbent structure. Additionally, a reduction in the aggregation of NiFe_2_O_4_ NPs is observed as they are dispersed within the chitosan solution during the synthesis of the sorbent. The image reveals that the structure of the sorbent is not uniform, with chitosan nanoparticles generally forming as rod-shaped particles positioned on the surface of NiFe_2_O_4_ NPs in certain areas. The TEM images indicated a uniform distribution of Cs@NiFe_2_O_4_ NPs with a typical size of 25 ± 5.05 nm. This finding confirms the potential of the developed composite material as an effective platform for a wide range of applications^42,56^.

### BET analysis

**Figure 7:**
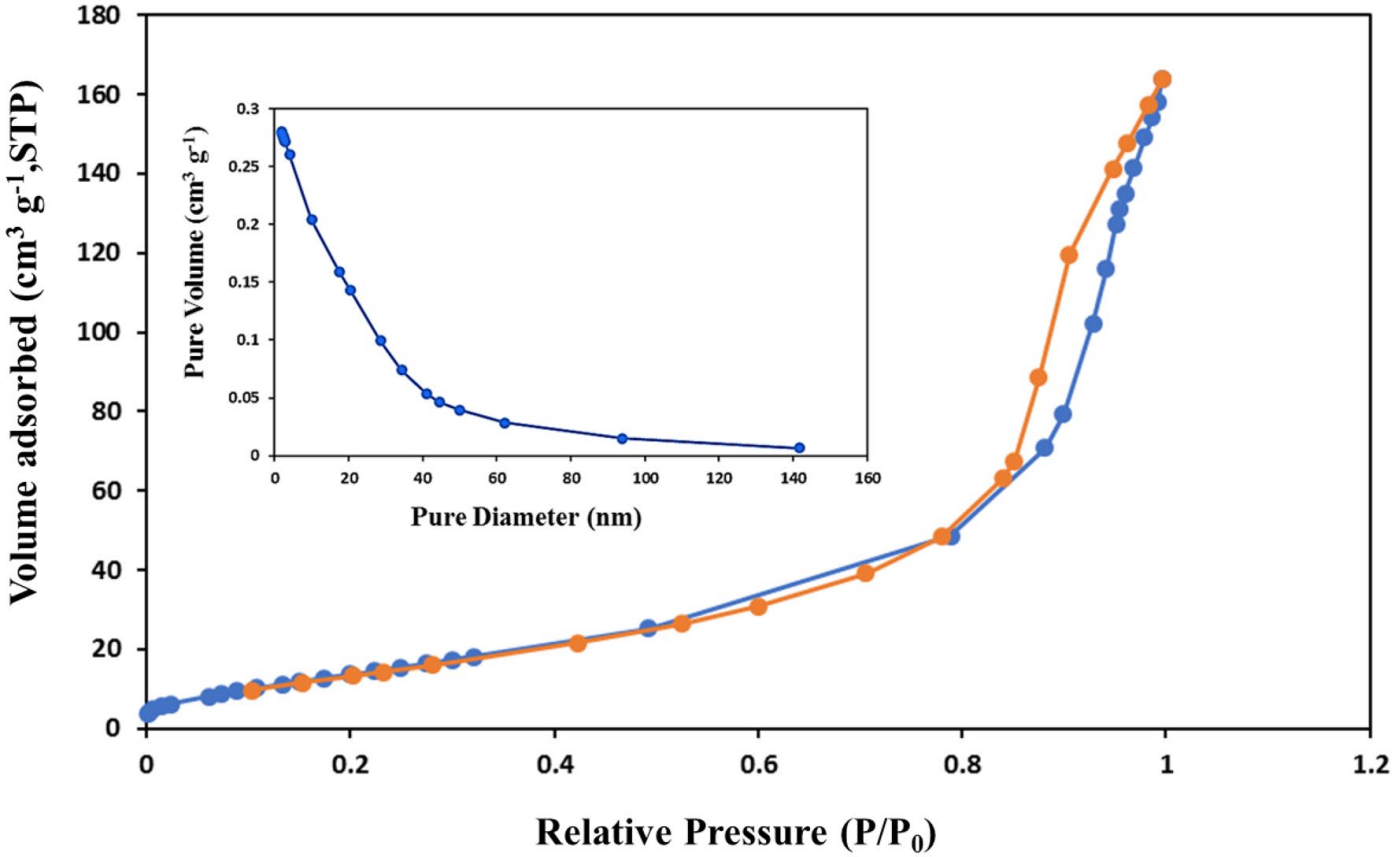
The BET image of Cs@NiFe_2_O_4_ NPs.

Accurate surface area and porosity measurement is essential for many applications, including catalysts, nano-sorbents, chemicals and additives, pharmaceuticals, and Food-related companies, as well as in nano-structures like metal nanoparticles, nano-tubes, nano-fibers, etc. Determination of the molecule's surface area helps to calculate the surface occupied by the molecule, the sorbent amount, and the total sample area. The surface area of the synthesized Cs@NiFe_2_O_4_ NPs was measured using the BET test^57^. Based on the results, the surface area of the particle was 59.134 m^2^ g^−1^, the medium pore diameter was 13.22 nm, the micropore volume was 0.279 cm^3^ g^−1^, and the porosity was meso-type (Fig. 7).

The type IV isotherm was obtained for the nanocomposite. This type of isotherm is used for porous materials and industrial catalysts, indicating the porous structure of the nanocomposite. Moreover, it demonstrates that the composite has very narrow pores in the capillary form, significantly enhancing the adsorption and the condensing of the sorbent on the surface. The corresponding curve was utilized to determine the pore size distribution ^57,58^.

### Modeling the sorption process

The impacts of several factors, including contact time, sorbent amount, pH, and dye concentration, were investigated on the removal efficiency of sunset yellow dye to maximize effectiveness of dye removal.

A two–level CCD comprising 30 runs of experiments on a block with a random run order was utilized to eliminate uncontrolled factors’ effects. Each experiment was carried out three times, and the average adsorption amount was utilized to calculate the removal efficiency. The designed experiments and the results are presented in Table 1.

The one-way ANOVA test with a confidence interval of 95% was used to evaluate the effect of each factor (Table 2). The analysis of variance demonstrated a high significance of the presented polynomial regression model to predict sunset yellow adsorption on Cs@NiFe_2_O_4_ NPs (*P* ˂ 0.05). Moreover, The *p*-value for lack of fit (LOF) indicates that the proposed model accurately describes experimental responses. The R-squared and adjusted R-squared (93.07 and 96.42, respectively) indicated a significant ability of the model to predict variations. Besides, the low standard deviation (4.22) and coefficient of variation (5.64%) represented a high accuracy and low error of the experiments.

**Table 2:** The ANOVA test for sunset yellow adsorption on Cs@NiFe_2_O_4_ NPs.

Source	DF	*P*-value
Model	14	**0.000 (significant)**
Blocks	1	0.000
Linear	4	0.000
F_1_	1	0.047
F_2_	1	0.010
F_3_	1	0.000
F_4_	1	0.000
Square	4	0.000
F_1_* F_1_	1	0.014
F_2_* F_2_	1	0.042
F_3_* F_3_	1	0.912
F_4_* F_4_	1	0.006
2-Way Interaction	6	0.000
F_1_* F_2_	1	0.011
F_1_* F_3_	1	0.193
F_1_* F_4_	1	0.328
F_2_* F_3_	1	0.000
F_2_* F_4_	1	0.022
F_3_* F_4_	1	0.029
Error	14	
LOF	10	**0.1018 (not significant)**
Pure error	5	
Total	29	

Significant values are in [bold]

**Figure 8:**
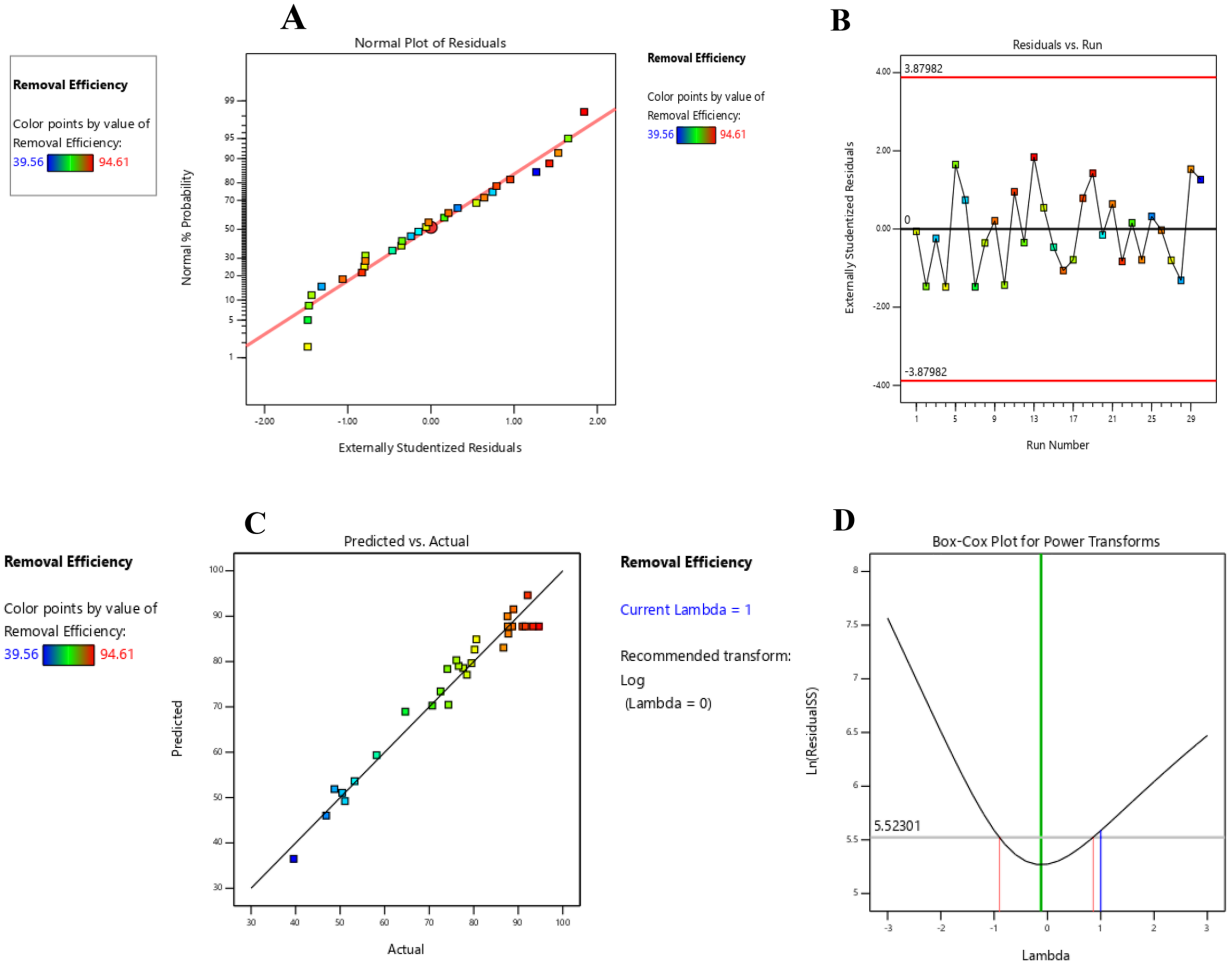
The normal probability (**A**), random distribution of residuals (**B**), predicted values versus experimental values (**C**), and Box-Cox (**D**) plots.

In the normal probability diagram, there are more points around the base line, which indicates that the curve follows the normal distribution (Fig. 8A).

The error diagram of the residuals displayed the random distribution of the data around the baseline, indicating that the results do not follow a specific pattern, which reflects the adequacy of the proposed response surface model to remove the sunset yellow dye (Fig. 8B). The Fig. 8C exhibited the consistency of the predicted and experimental results. The Box-Cox diagram (Fig. 8D) indicated the normal distribution of the data, representing the adequacy of the proposed model in removing the dye.

Table 2 shows the significant effect of all the factors and the interactions F_4_*F_4_, F_1_*F_1_, and F_2_*F_2_ on the removal efficiency of sunset yellow dye, with F_4_*F_4_ as the most significant. Four two-way interactions between dye concentration and sorbent amount (F_1_*F_2_), sorbent amount and contact time (F_2_*F_3_), sorbent amount and pH (F_2_*F_4_), and pH and contact time (F_3_*F_4_) indicated a significant effect on sunset yellow removal and other two-way interactions were not considerable for dye removal.

Regression analysis can be calculated using the central composite design to express the relationship between significant variables and experimental responses. Based on the findings, a full quartic mathematical model is the most effective way to determine this relationship, as shown by the following Eqs. 3.

3 $${\text{Removal Efficiency }}\left( \% \right) \, = { 2}.{\text{14F}}_{{1}} + { 2}.{\text{93F}}_{{2}} + { 7}.{\text{16F}}_{{3}} - { 1}0.{\text{51F}}_{{4}} + {3}.0{\text{5F}}_{{1}} {\text{F}}_{{2}} - { 1}.{\text{43F}}_{{1}} {\text{F}}_{{3}} + { 1}.0{\text{7F}}_{{1}} {\text{F}}_{{4}} + {7}.{\text{34F}}_{{2}} {\text{F}}_{{3}} + {2}.{\text{86F}}_{{2}} {\text{F}}_{{4}} + { 2}.{\text{53F}}_{{3}} {\text{F}}_{{4}} {-}{ 7}.{\text{26F}}_{{1}}^{{2}} {-}{ 5}.{\text{79F}}_{{2}}^{{2}} {-} \, 0.{\text{2934F}}_{{3}}^{{2}} {-}{ 8}.{\text{29F}}_{{4}}^{{2}} + { 87}.{76}$$

### Effect pH

**Figure 9:**
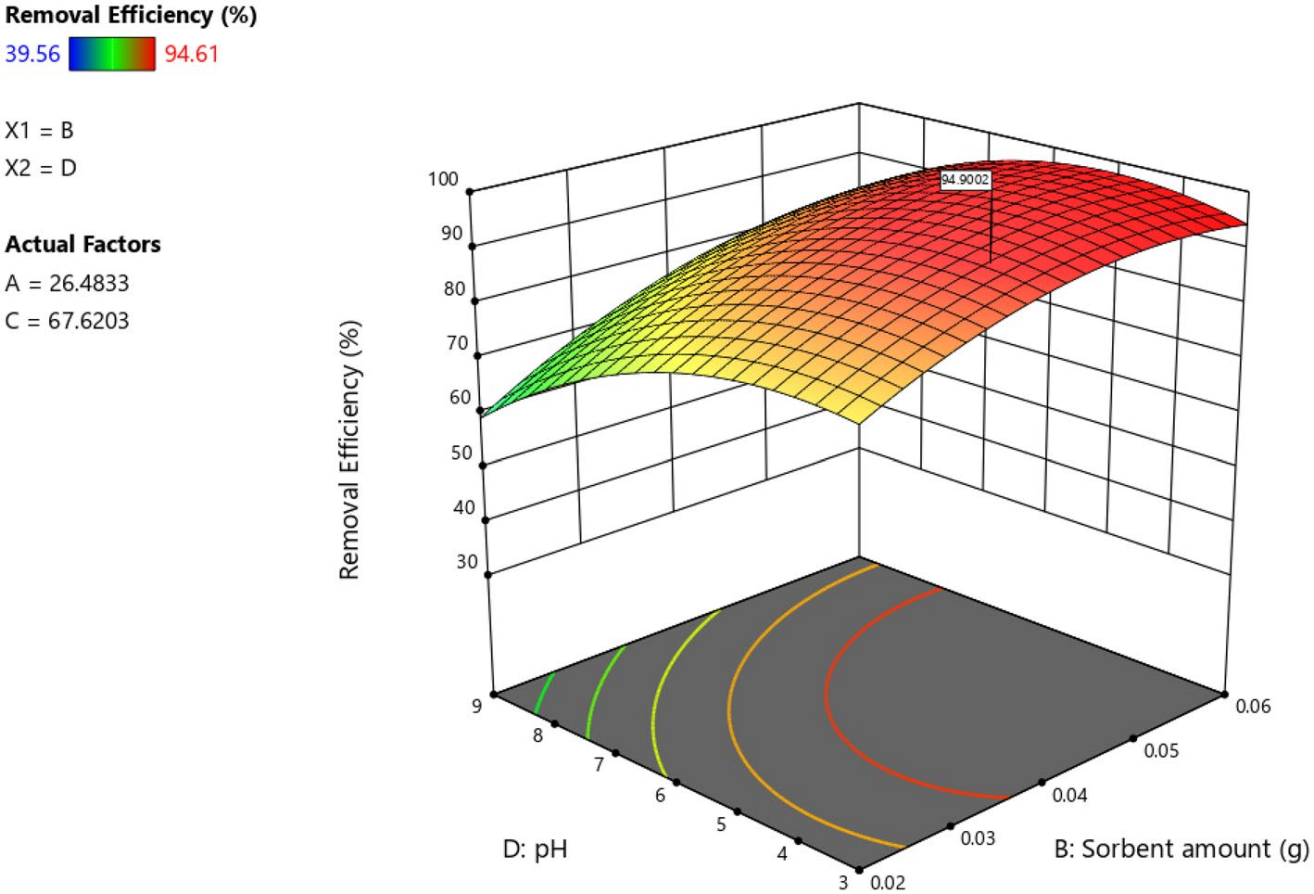
The interaction of sorbent and pH.

In this study, we looked into the effect of pH in the range of 3–9 on dye adsorption by Cs@NiFe_2_O_4_ NPs. Figure 9 shows the impact of pH and sorbent amount interaction about the adsorption of azo dye molecules by the synthesized nano-sorbent. The results indicated that decreasing pH led to a significant increase in adsorption capacity of the sorbent and efficiency of dye removal. Two processes may occur for adsorbing the anionic dye onto the sorbent. First, the sunset yellow dye penetrates the sorbent pores through physical adsorption. Therefore, the sorbent components chitosan and magnetic Fe_2_O_4_ significantly impact the process. Second, it is due to intermolecular links (O–H⋯N, O–H⋯π) and electrostatic interactions between the positive surface charge of Cs@NiFe_2_O_4_ NPs and the negative charge of the anionic dye.

It has been indicated that raising the pH to ˃7 results in a negative charge on the sorbent surface, and at lower pHs, a positive surface charge will be created. The Sunset yellow is an anionic compound. Therefore, enhancing electrostatic attraction in the middle of the sorbent and the dye is the main factor in increasing adsorption capacity. At acidic pHs, the amine groups of chitosan will be protonated, increasing the electrostatic force between the negatively charged dye molecules and the positively charged sorbent active sites. Under alkaline conditions, the production of hydroxide functional groups in the environment leads to a negative charge on the sorbent surface, resulting in a weak interaction or even a repulsive electrostatic force between the anionic dye molecule and the sorbent, decreasing the adsorption amount. The results were consistent with those of Travolo et al.^59^ and Aliabadi et al.^60^.

### Sorbent amount, dye concentration, and contact time

**Figure 10:**
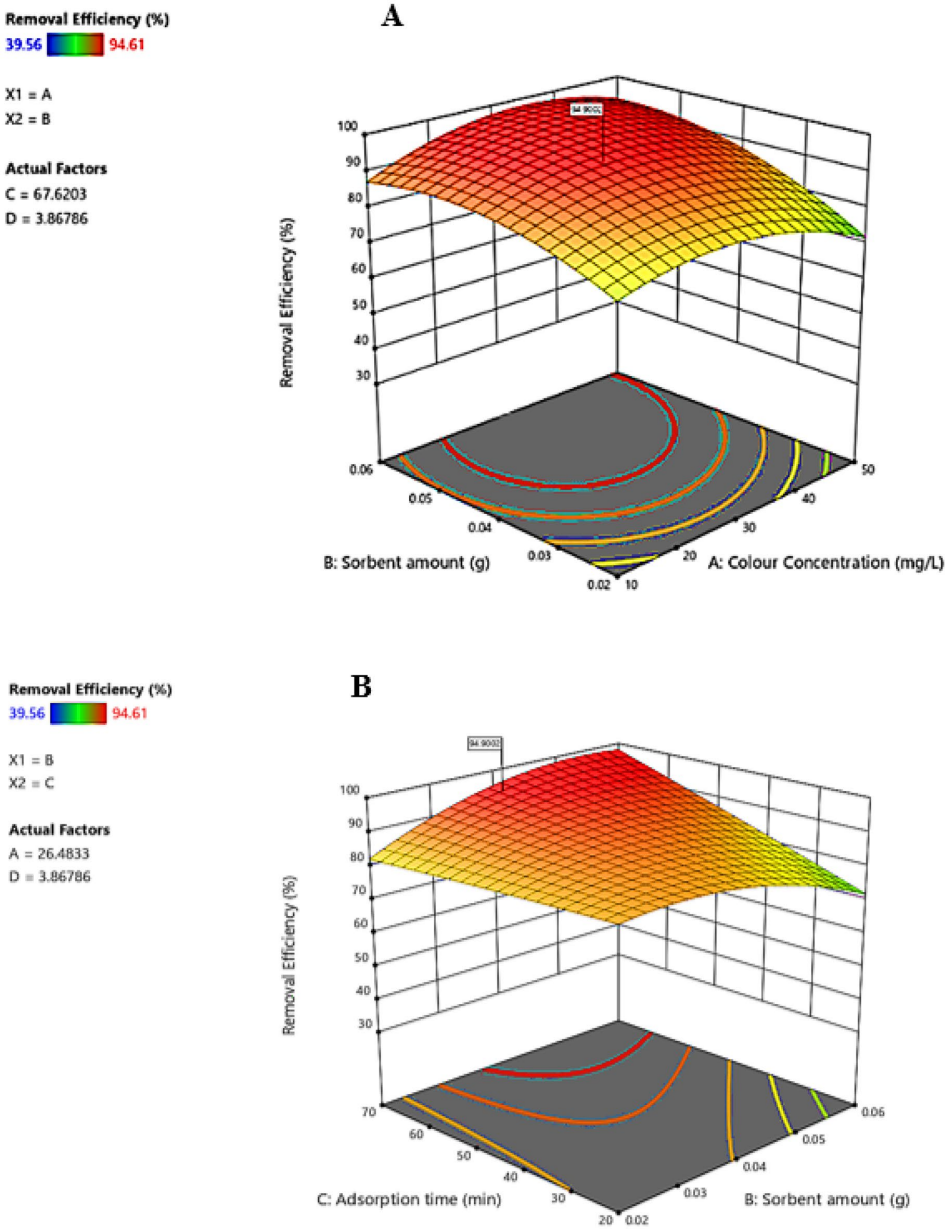
The interactions of sorbent dose and dye concentration (**A**) and sorbent dose and contact time (**B**).

Increasing the dosage of sorbent and reducing the initial dye concentration resulted in a higher removal percentage (Fig. 10A). The explanation for this is that at low dye concentrations, and high sorbent dosage, more active sites are available to adsorb a higher dye percentage. Increasing initial dye concentration decreases the removal percentage due to filling adsorption sites at higher concentrations. Moreover, it resulted in a higher dye adsorption capacity by increasing the driving force for mass transfer on the sorbent surface.

Increasing the sorbent amount and contact time enhanced the dye removal efficiency (Fig. 10B). The color adsorption on the sorbent is maximum in the first minutes due to the high contact surface and accessibility of the sorbent sites. After equilibrium, it decreases by the saturation of surface active sites of the sorbent. Therefore, the sorbent is no longer available to remove the color. Results were in line with the studies of Roosta et al.^61^, Sadegh et al.^62^.

### Adsorption isotherm

Adsorption isotherm is an important factor in the design of absorption systems and describes the interaction between the sorbent and the adsorbed material. Therefore, it is always considered substantial for determining the capacity for adsorption of a sorbent and optimizing the sorbent amount. The data on equilibrium obtained to aid in the absorption of sunset yellow in the concentration range of 10–150 mg L^−1^ on the sorbent (0.045 g L^-1^) by the Langmuir and Freundlich isotherms (Table 3) were used to match the experimental data, which represent information about the system and the capability for adsorption of the sorbent. The linear versions of the Langmuir and Freundlich equations (Eqs. 4 and 5, respectively) are as follows^63–65^.

**Table 3:** Adsorption kinetics and isotherms for sunset yellow removal by Cs@NiFe_2_O_4_ NPs.

Kinetic model	Equation	Parameters		R^2^
Pseudo- first- order model	${\text{log}}\, \left({q}_{e}-{q}_{t}\right)= {\text{log}}{q}_{e}- \frac{{k}_{1}}{2.303}t$	q_e_ (mg g^-1^)	95.19	0.9594
		K_1_ (min^-1^)	0.011	
Pseudo- second- order model	$\frac{t}{{q}_{t}}=\frac{1}{{k}_{2}{q}_{e}^{2}}+\frac{1}{{q}_{e}}t$	q_e_ (mg g^-1^)	109.89	0.9895
		K_2_ (g mg^-1^ min^-1^)	0.000203	
Intra-particle diffusion model	${q}_{t}={k}_{i }* {t}^\frac{1}{2}+C$	K_i_ (mg g^−1^ min^−1/2^)	8.76	0.9755

Isotherm model	Equation	Parameters		R^2^
Langmuir model	$\frac{{{\text{C}}}_{e}}{{q}_{e}}=\frac{{C}_{e}}{{q}_{max}}+\frac{1}{{bq}_{max}}$	q_max_ (mg g^-1^)	212.766	0.9682
		b (L mg^-1^)	0.049	
Freundlich model	${{\text{ln}}q}_{e}={{\text{ln}}k}_{f}+\frac{1}{n}{{\text{ln}}C}_{e}$	K_f_ (mg g^-1^)	18.60	0.9888
		n	1.80	

4 $$\frac{{{\text{C}}}_{e}}{{q}_{e}}=\frac{{C}_{e}}{{q}_{max}}+\frac{1}{{bq}_{max}}$$

5 $${{\text{ln}}q}_{e}={{\text{ln}}k}_{f}+\frac{1}{n}{{\text{ln}}C}_{e}$$

Where C_e_ and q_e_ are the initial concentration of the dye in the sample solution and the equilibrium adsorption amount per sorbent mass (mg g^−1^), respectively. The q_max_, b, k_f_, and n are the maximum monolayer adsorption capacity (mg g^−1^), Langmuir constant (mg L^−1^), maximum Freundlich adsorption capacity (mg g^−1^), and adsorption intensity, respectively.

**Figure 11:**
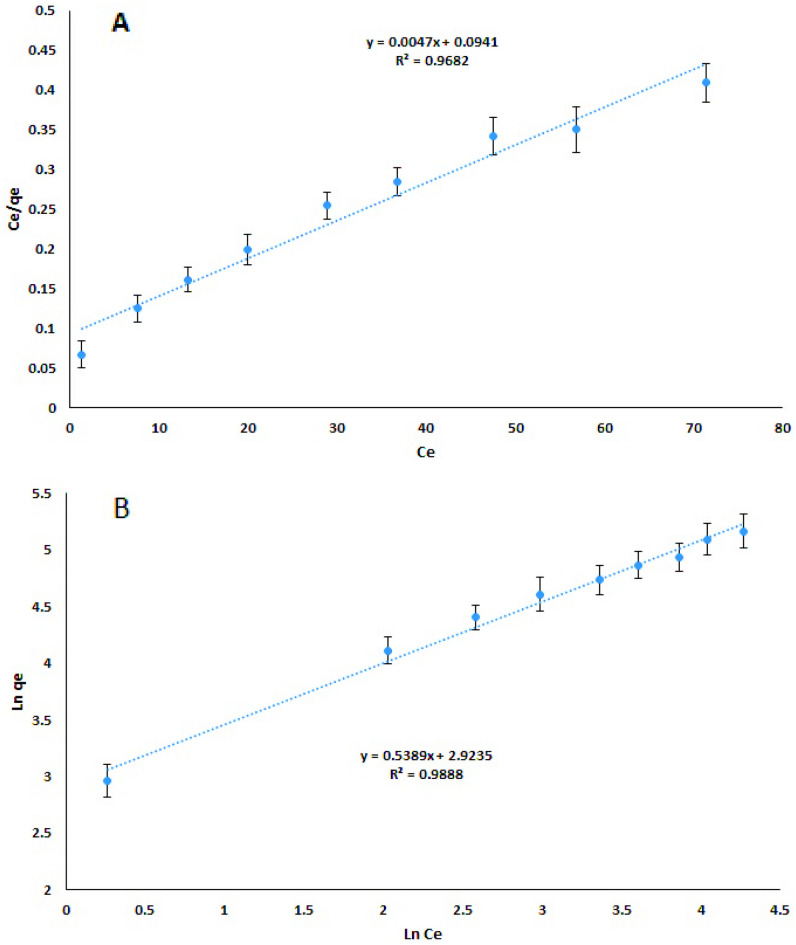
Langmuir (**A**) and Freundlich (**B**) sorption diagrams (n = 3).

The graphs of C_e_/q_e_ versus C_e_ and ln q_e_ versus ln C_e_ were drawn to determine the values of the parameters and determine the appropriate isotherm model using the regression coefficient (R^2^). According to the findings, Freundlich isotherm (Fig. 11B) can better predict dye adsorption by Cs@NiFe_2_O_4_ NPs than Langmuir isotherm (Fig. 11A). Therefore, it was concluded that the sorbent surface is heterogeneous, and adsorption is mainly performed in several layers. These outcomes matched up with what was seen in the sorbent's SEM photos.

According to the Freundlich equation, the adsorption intensity (n) is usually utilized to evaluate the desirability of the process of adsorption. Optimal adsorption happens when the value of n is in the range of 1 to 10. The attained value of n is 1.85, indicating the process of adsorption appropriateness. The highest adsorption capacity of sunset yellow dye on Cs@NiFe_2_O_4_ NPs (q_max_) was determined using the Langmuir equation's slope as 212.766 mg g^−1^.

### Adsorption of kinetics

**Figure 12:**
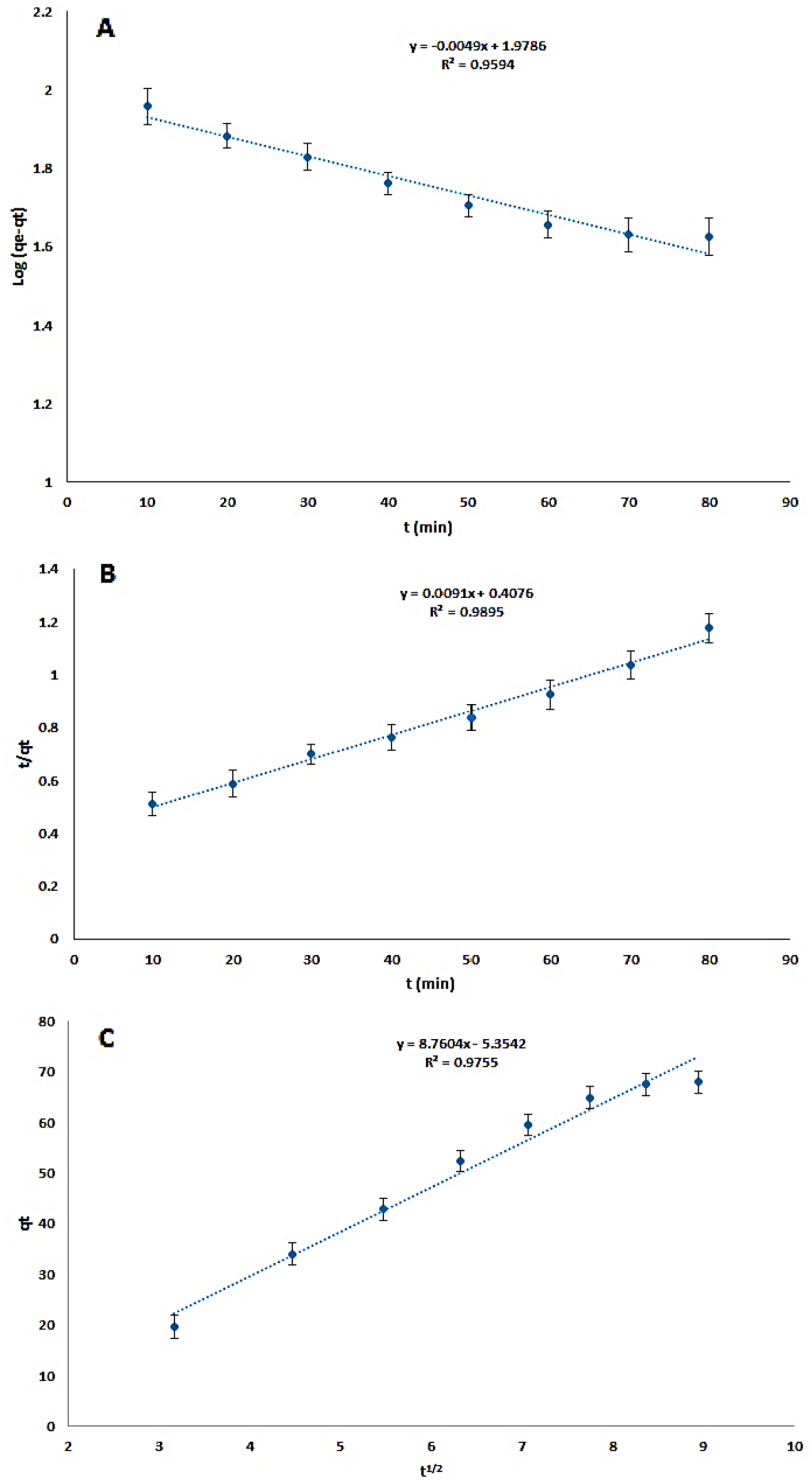
The effect of contact time on sunset yellow removal by the CS@ NiFe_2_O_4_ NPs (n = 3): The pseudo-first-order kinetic model (**A**), pseudo-second-order kinetic model (**B**), and intraparticle diffusion model (**C**).

Adsorption kinetics were looked into to understand the dynamics of dye adsorption on Cs@NiFe_2_O_4_ NPs due to the abundance of functional groups, such as an increased surface-to-volume ratio, oxygen, and to prepare a predictive model that allows estimating the amount of dye adsorbed during the process. This information can be used to design large systems. Therefore, model kinetics, including pseudo-first-order, pseudo-second-order, and intraparticle diffusion, were extensively used to examination of adsorption process kinetics and fitness of the data on adsorption versus time (Table 3). The linear forms of pseudo-first-order, pseudo-second-order, and intraparticle diffusion models (Eqs. 6, 7, and 8, respectively) for use in the kinetic study are as follows^66^.

6 $${\text{log}}\,\left({q}_{e}-{q}_{t}\right)= {\text{log}}{q}_{e}- \frac{{k}_{1}}{2.303}t  $$

7 $$\frac{{\text{t}}}{{q}_{t}}=\frac{1}{{k}_{2}{q}_{e}^{2}}+\frac{1}{{q}_{e}}t$$

8 $${q}_{t}={k}_{i }* {t}^\frac{1}{2}+C$$

where k, q_e_ (mg g^−1^), and q_t_ (mg g^−1^) are the rate constant, the amount of dye adsorbed at equilibrium, and that adsorbed at time t (min), respectively. The impact of contact time in the range of 10–80 min under optimal conditions was investigated to evaluate the kinetic models’ capability to estimate the dye adsorption amount by Cs@NiFe_2_O_4_ NPs (Fig. 12).

The kinetic model of pseudo-first order states that the penetration occurs inside the monolayer, and the variations of the adsorption amount versus time are in proportion to quantity of vacant sorbent sites. The kinetic model of pseudo-first order expresses retarding chemical adsorption process that controls the adsorption, in which the square of the number of unoccupied sites in the sorbent is proportional to the occupation rate of the adsorbed sites.

A linear relationship is obtained by plotting log (q_e_-q_t_) versus t, and k_1_ and q_e_ can be determined from the line slope and y-intercept, respectively. If the y-intercept is not equal to q_e_, the reaction does not resemble a first-order reaction, even if the diagram has a high correlation coefficient.

Experimental findings for the pseudo-first-order kinetic model (Fig. 12A) and intraparticle diffusion model (Fig. 12C) demonstrated a high degree of non-linearity and a low correlation coefficient.

The pseudo-second-order kinetic diagram (Fig. 12B) obtained by plotting the t/q_t_ values versus t resulted in a straight line with a high correlation coefficient. The second-order kinetic velocity constant (k_2_) and equilibrium adsorption capacity (q_e_) were calculated from the y-intercept and the slope of the t/q_t_ graph vs. t, respectively. R^2^ and q_e_ values demonstrated that the model of kinetic pseudo-second order leads to better results.

The removal efficiency (%) increased up to 68 min and at higher times remaining practically constant. Consequently, the adsorption process is rapid and can achieve equilibrium in under 70 min. This process is mainly performed as a result of the interaction of the anionic dye with the functional groups on the sorbent surface. In addition, low values for the correlation coefficient for the intraparticle distillation model confirmed that the dye diffusion in the pores of the sorbent is notably low, and the sunset yellow adsorption is caused by physical or chemical interactions with CS@NiFe_2_O_4_ NPs (Fig. 12, Table 3).

### Removal of dye from orange juice samples

The dye removal efficiency of 91.75% and 93.24% was obtained using the nanocomposites for industrial and traditional orange juices, respectively. The outcomes demonstrated that the nanoparticles have acceptable efficiency in the surface adsorption of sunset yellow from the fruit juice samples on a laboratory scale.

### Recovery capability

**Figure 13:**
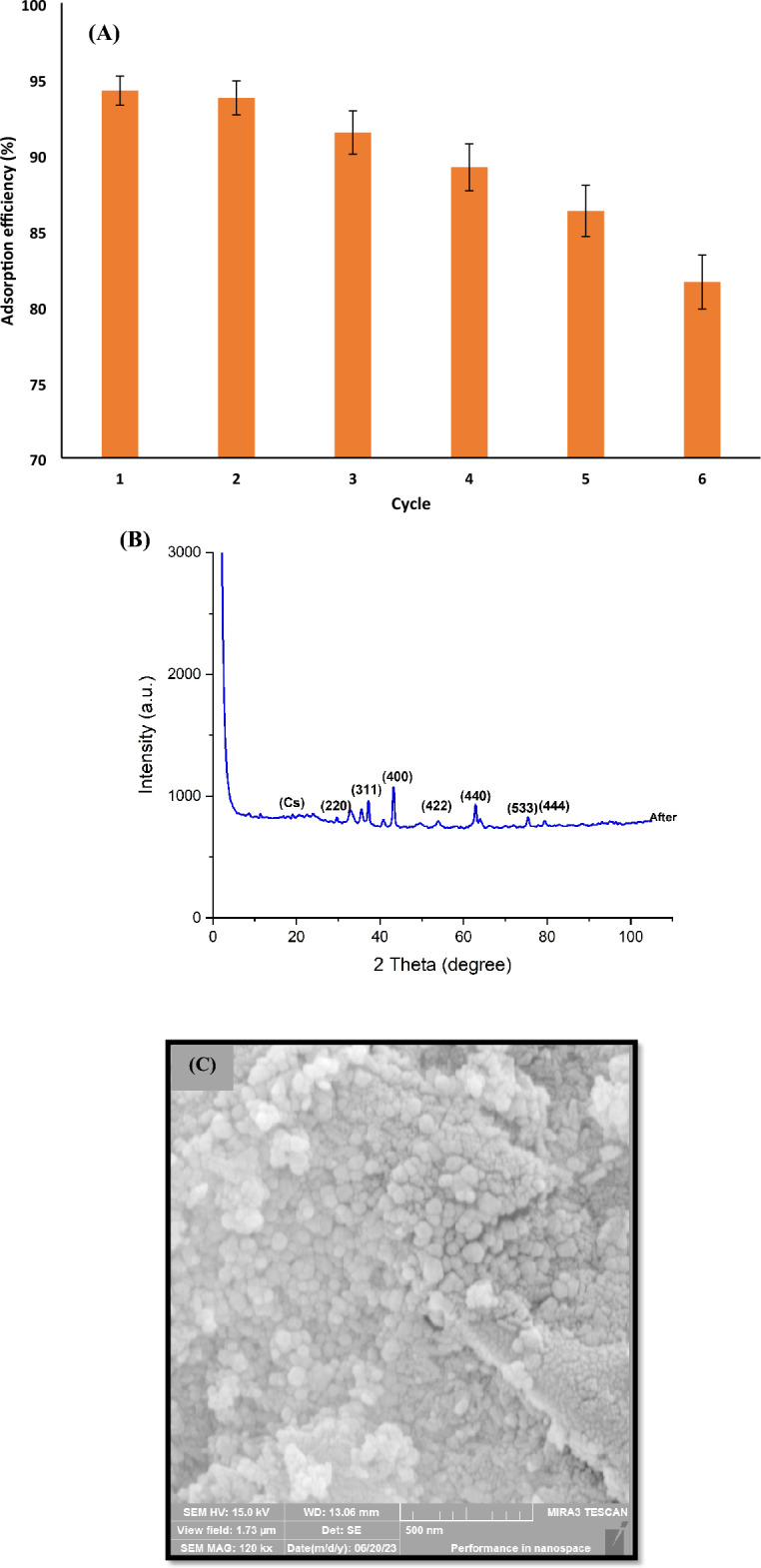
Reusability of Cs@NiFe_2_O_4_ NPs for sunset yellow removal (n = 3) (**A**), XRD pattern (**B**) and SEM image (**C**) of Cs@NiFe_2_O_4_ NPs after five adsorption–desorption cycles.

The recoverability of the sorbent is economically significant, indicating the cost-effectiveness of using the sorbent in pollutant removal, the constancy of the adsorbed material on the sorbent surface, and the sorbent regeneration conditions. After the removal procedure, the sorbent was washed using distilled water and then separated using a magnet. An aqueous ammonium solution (0.2 M, 1.0 mL) was added to the sorbent and shaken for 10 min at 150 rpm. The sorbent was then washed with distilled water before reuse. The results in (Fig. 13A) demonstrated that the adsorption efficiency of sunset yellow dye by Cs@NiFe_2_O_4_ NPs did not significantly change up to 5 cycles and was above 86%. Any decline in the effectiveness of dye adsorption could be attributed to the inadequate separation of the dye from the sorbent, the slight loss of sorbent during its separation from the solution, and the degradation of the sorbent structure during the dye removal step. Consequently, the synthesized nanocomposite is an effective and enduring sorbent for sunset yellow dye removal, consistent with the findings of Ansari et al.^56^ and Habiba et al.^67^. To evaluate the change in the sorbent structure after the desorption process, the XRD pattern of the sorbent after five adsorption–desorption cycles is shown in Fig. 13B, indicating no significant change in the sorbent structure. Only a slight decrease in the intensity of the peaks in this pattern compared to the initial pattern of the sorbent was observed. The SEM image (Fig. 13C) clearly shows that pores remained on the surface of Cs@NiFe_2_O_4_ NPs, indicating that less pores may be saturated by the dye sunset yellow during the adsorption process.

### Comparison with other sorbents

Table 4 presents a comparison of the efficiency of the synthesized sorbent with other sorbents previously studied for the removal of sunset yellow dye. The data indicates that the developed sorbent demonstrates a favorable adsorption capacity, surpassing most of the prepared sorbents. While sorbent No. 2 (PDMAEMA grafted PS-DVB-VBC) exhibits a higher absorption capacity than the synthetic sorbent, it necessitates a relatively low acidic pH and a larger quantity of sorbent to achieve dye removal. Likewise, the use of sorbent No. 6 (Alligator weed activated carbon) and No. 8 (Thermally reduced graphene oxide) for sunset yellow dye removal requires a significantly longer contact time and a higher amount of sorbent compared to the sorbent prepared in this study, respectively.

**Table 4:** Comparison of the various sorbents for sunset yellow removal.

No	Sorbent	Qe (mg g^−1^)	pH	sorbent dosage (g L^−1^)	dye concentration (mg L^−1^)	Time (min)	Ref
1	Activated carbon	44.9	1–2	0.1	150	90	^68^
2	PDMAEMA grafted PS-DVB-VBC	312.5	2	0.4	20	40	^69^
3	OMC-2Nd	285	6.5	0.1	50	240	^70^
4	PPy/mw nanocomposite	212.1	2	0.007	50	5	^60^
5	MAGO/CTAB	44.2	6	0.02	30	20	^71^
6	Alligator weed activated carbon	271	3	0.8	150	240	^72^
7	Nickel ferrite	107.1	3	–	–	–	^73^
8	TRGO	243.3	6	0.25	25	30	^74^
9	m–CS–c–PAM	359.71	2–10	1	20	60	^58^
10	Cu; ZnS-NP-AC	85.39	6	0.025	20	3	^75^
11	ZnO NPs loaded on activated carbon	142.8	2	0.015	20	10	^76^
12	CS@NiFe_2_O_4_	212.76	3.87	0.038	26.48	67.62	This work

Study No. 9, the sorbent (Magnetic Fe_3_O_4_ embedded chitosan–crosslinked-polyacrylamide composites) demonstrates superior efficiency for dye removal compared to the prepared sorbent. However, it is noteworthy that the steps, reagents, and synthesis time required for preparing this sorbent are significantly greater than those for the sorbent in the study. Additionally, the use of synthetic polymer compounds in this sorbent, while enhancing its stability, could potentially lead to environmental concerns due to their persistence. Therefore, the synthesized sorbent exhibits the proper ability to remove sunset yellow dye with suitable reusability, adsorption capacity, and contact time, making it a promising candidn ate for the effective removal of this dye from food samples.

## Conclusion

Cs@NiFe_2_O_4_ NPs, a new magnetic adsorbent, were created and characterized using various techniques. Cs@NiFe_2_O_4_ NPs is a green and efficient sorbent for the sunset yellow removal dye from food samples. The importance of Cs@NiFe_2_O_4_ NPs as a sorbent for removal of synthetic dyes lies in their high adsorption capacity, regeneration potential, environmental friendliness, and fast adsorption rate, making them a promising solution for addressing the challenges of synthetic dye removal in industrial settings. The process of adsorption's main contributing variables, such as pH, contact time, dye concentration, and sorbent amount, were carefully tuned using a design experiment methodology. The sunset yellow dye was adsorbed to the developed nanocomposite mainly by electrostatic interactions. Adsorption data were matched with Freundlich isotherm and models of pseudo-second-order kinetics. The produced sorbent showed excellent efficiency for the recovery of sunset yellow dye, which was greater than 86% after five cycles of adsorption and desorption. The recovery data for juice samples indicated that the synthesized nanocomposite could be an efficient sorbent for sunset yellow removal in food samples.

## Data Availability

The datasets generated and analyzed during the current study were available from the corresponding author on reasonable request.
