# Author Correction: Modeling sunset yellow removal from fruit juice samples by a novel chitosan-nickel ferrite nano sorbent

**DOI:** 10.1038/s41598-024-55343-8

**Published:** 2024-02-27

**Authors:** Samira Shokri, Nabi Shariatifar, Ebrahim Molaee-Aghaee, Gholamreza Jahed Khaniki, Parisa Sadighara, Mohammad Ali Faramarzi

**Affiliations:** 1 Department of Environmental Health Engineering, Food Safety Division, School of Public Health, Tehran University of Medical Sciences, Tehran, Iran; 2 Department of Pharmaceutical Biotechnology, Faculty of Pharmacy, Tehran University of Medical Sciences, Tehran, Iran

Correction to: *Scientific Reports* 10.1038/s41598-023-50284-0, published online 02 January 2024

The Acknowledgements section in the original version of this Article was incomplete.

“The authors acknowledge the financial and technical support of the Tehran University of Medical Sciences, Tehran, Iran (Grant Number: 62414; Ethics Code: IR.TUMS.SPH.REC.1401.157).”

now reads:

“The authors acknowledge the financial and technical support of the Tehran University of Medical Sciences, Tehran, Iran (Grant Number: 62414; Ethics Code: IR.TUMS.SPH.REC.1401.157). This work is based upon research funded by Iran National Science Foundation (INSF) under project No.4025197.”

The original Article has been corrected.

